# The Innocent Victims of a Conscience Clause

**Published:** 1902-05-03

**Authors:** 


### THE INNOCENT VICTIMS OF A CONSCIENCE CLAUSE.

An interesting summary of the conditions as to vaccination found in the 1,015 cases in which death from small-pox had occurred during the present epidemic among London residents has been recently issued by the Registrar-General, and the facts therein set forth serve well to demonstrate the protective powers of vaccination for at least some years after its performance. To put the matter shortly it may be said that, if we include in one great group the deaths which occurred among the vaccinated, and—to prevent any possible protest by the anti-vaccinists—all the cases in which there was any doubt as to whether they had been vaccinated or not, these deaths numbered 564, of which only four were children under 10 years of age. If, however, we include in another great group all the deaths which occurred in unvaccinated cases, and all those which occurred among persons not vaccinated until after they were infected by small-pox, we find that these deaths numbered 451, of which 264 were in children under 10 years of age. We need hardly say that one of the strong proofs of the efficacy of vaccination is derived from the fact that since the introduction of that measure the age incidence of small-pox has completely changed; the disease in old days having been specially fatal among the young, while now it is one of the very rarest of events to find a death from small-pox in a vaccinated child under ten years of age. The anti-vaccinists have been by no means blind to this difficulty, and various more or less ingenious explanations have been put forward to explain a fact which was incontrovertible. The ablest of the anti-vaccinists have held that this change in the age incidence was to be explained by the fact that by the abolition of the practice of inoculation, by which sources of small-pox infection were constantly maintained, and by the improved methods now employed to prevent the spread of the disease, the length of the interval between successive epidemics of small-pox had become so much more prolonged that a larger proportion of the population had grown up to higher ages in a state of susceptibility. These statistics, however, are entirely free from all those fallacies which may be imagined to attach to a comparison of one period with another. Here we have two groups of persons differentiated solely by the fact that the members of one group have been vaccinated and those of the other have not, and they are attacked in the same epidemic by an infection apparently of similar intensity. We have nothing to do with the proportion of one group to the other, or the proportion in either one or the other which was attacked by small-pox; all we are concerned with is the way in which the disease fell upon the different ages within each group. In the unvaccinated group, which we may take as representing the natural history of the disease, we find that more than half the victims were under ten years old, whereas in the vaccinated group not one per cent. of the deaths were in children under 10. What then is the difference between these two groups that leaves the one group practically untouched by the disease while the other suffers so heavily, unless it be that the children in the one had been vaccinated and in the other not? This is what has to be answered by those who pretend to disbelieve in the efficacy of vaccination. It is to be noted, moreover, that for the sake of meeting all possible objections, we have stated the case in a very extreme form, for if we had removed from the vaccinated class those cases in which there was a doubt as to whether vaccination had been done or not, and one case in which it was known that vaccination had been imperfectly performed, we should have had in the whole of this series of 1,015 deaths from small-pox not one single instance of a vaccinated child under 10 years of age dying of the disease, while considerably more than half of all the deaths among unvaccinated persons were those of children under 10, children who could be differentiated in no other way from that group, not one of whom died except by the fact that they were either unvaccinated or had not been vaccinated until after they had been infected by small-pox. Certainly the Registrar-General is to be thanked for publishing such an interesting statistical summary.

